# Detection of oocyte perivitelline membrane-bound sperm: a tool for avian collection management

**DOI:** 10.1093/conphys/cou060

**Published:** 2015-01-19

**Authors:** Kaitlin E. Croyle, Barbara S. Durrant, Thomas Jensen

**Affiliations:** 1 San Diego Zoo Institute for Conservation Research, 15600 San Pasqual Valley Road, Escondido, CA 92027, USA; 2 California State University San Marcos, 333 S. Twin Oaks Valley Road, San Marcos, CA 92096, USA

**Keywords:** Avian conservation, breeding management, perivitelline membrane, sperm

## Abstract

The success and sustainability of an avian breeding programme depend on managing productive and unproductive pairs. Given that each breeding season can be of immeasurable importance, it is critical to resolve pair fertility issues quickly. Such problems are traditionally diagnosed through behavioural observations, egg lay history and hatch rates, with a decision to re-pair generally taking one or more breeding seasons. In pairs producing incubated eggs that show little or no signs of embryonic development, determining fertility is difficult. Incorporating a technique to assess sperm presence on the oocyte could, in conjunction with behaviour and other data, facilitate a more timely re-pair decision. Detection of perivitelline membrane-bound (PVM-bound) sperm verifies successful copulation, sperm production and sperm functionality. Alternatively, a lack of detectable sperm, at least in freshly laid eggs, suggests no mating, lack of sperm production/function or sperm–oviduct incompatibility. This study demonstrated PVM-bound sperm detection by Hoechst staining in fresh to 24-day-incubated exotic eggs from 39 species representing 13 orders. However, a rapid and significant time-dependent loss of detectable PVM-bound sperm was observed following incubation of chicken eggs. The PCR detection of sperm in seven species, including two bacterially infected eggs, demonstrated that this method was not as reliable as visual detection using Hoechst staining. The absence of amplicons in visually positive PVMs was presumably due to large PVM size and low sperm count, resulting in DNA concentrations too low for standard PCR detection. In summary, this study demonstrated the feasibility and limitations of using PVM-bound sperm detection as a management tool for exotic avian species. We verified that sperm presence or absence on fluorescence microscopy can aid in the differentiation of fertile from infertile eggs to assist breeding managers in making prompt decisions for pair rearrangements. This protocol is currently used to manage several breeding pairs in San Diego Zoo global avian conservation programmes.

## Abstract (precis)

This study evaluated the use of nuclear staining and PCR to detect the presence of sperm on avian oocyte membranes following incubation, room temperature and 4oC storage, and bacterial infection. The technique can be used as part of the reproductive evaluation process and for managing birds in captive breeding facilities.

## Introduction

Captive propagation is often an essential component of endangered species recovery. Captive populations provide a genetic safeguard against extinction in the wild and play an important role in the management of threatened wild populations (Philippart, 1995). One of the main objectives for restoring species on the verge of extinction is maximization of reproductive output; every breeding opportunity is crucial.

Assisted reproductive technologies, such as artificial insemination and artificial incubation, are important tools for management of captive avian populations because they can help to increase fertility and hatch rates. Determination of the cause of infertility and offspring mortality is critical for maintaining a successful avian breeding programme; therefore, troubleshooting of pairing, incubation, developmental and hatching failure is common practice in zoos and captive-breeding facilities. Identification of infertility vs. early embryonic death may help in determining whether genetic defects, incubation behaviour or environmental factors are the cause of embryo mortality (Christensen, 2001). However, with the absence of a visible embryo, it can be difficult to distinguish between very early embryonic mortality and an unfertilized egg. While the germinal vesicle and blastoderm can be distinguished in freshly laid eggs (Kosin, 1945), they deteriorate during incubation, which makes fertility assessment increasingly difficult (T. Jensen, personal observation). While the presence of an embryo in an unhatched egg confirms breeding pair fertility, the egg with no visible embryonic growth raises questions and concerns about pair compatibility and/or individual infertility.

Additional methods of fertility assessment are required for incubated eggs that do not show signs of embryonic development upon candling and breakout. Avian perivitelline membrane (PVM) sperm detection was previously described by Bakst and Howarth (1977), Wishart (1987, 1997) and Bramwell and Howarth (1992). This method has been used in a variety of field studies to infer egg fertility (Kempenaers *et al.*, 1996; Birkhead *et al*., 2008), to demonstrate a decrease in PVM-bound sperm in successive eggs within a clutch (Birkhead *et al*., 1994) and lay cycles (Wishart, 1987), and to study the minimal number of sperm needed for fertilization (Wishart, 1987), the localization of sperm binding on the PVM (Bramwell and Howarth, 1992; Wishart, 1997; Rabbani *et al*., 2006), the PVM-bound sperm concentrations in fertile (Birkhead *et al*., 1993; Wishart, 1997; Malecki and Martin, 2003) and in failed clutches (Schut *et al*., 2014), sperm competition (Birkhead *et al*., 1995) and DNA and microsatellite isolation (Kempenaers *et al*., 1996; Carter *et al.*, 2000; Martínez and Burke, 2003).

While PVM-bound sperm staining has been used in field studies of several avian species, it has not been widely used for population fertility assessment or management of captive, non-domestic species. In fact, the potential for PVM-bound sperm staining in the assessment of breeding success in endangered species propagation has been proposed in only two previous reports, yielding conflicting results. Using domestic turkey eggs, Small *et al*. (2000) concluded the method to be of limited application, deeming avian sperm nuclei to be too fragile to be detected following 7 days of incubation. These results were contradicted in a study by Hemmings *et al.* (2012), which demonstrated that PVM-bound sperm in turkey eggs did not degrade significantly during incubation for up to 20 days.

These contradictory results and the possibility for application of this method to captive management of exotic birds led to the present study. The potential benefit of this method lies in its ability to rule out male fertility issues if sperm is present, and instead suggest problems with incubation behaviour, genetic compatibility or ovarian meiosis. In contrast, continual absence of sperm in undeveloped eggs suggests physiological incompatibility, behavioural incompatibility or male infertility, all of which might be distinguished by review of pair/individual breeding histories.

This study demonstrated that PVM-bound sperm detection can be used to evaluate and manage pairings for captive avian breeding and conservation programmes, through confirmation of sperm function following natural as well as artificial insemination. However, due to sperm degradation, membrane breakdown and bacterial or fungal contamination, the time window for accurate fertility assessment is limited, which may lead to false-negative interpretations. In this study, we established time and temperature parameters for post-oviposition sperm detection using unincubated and euthanized, incubated chicken (*Gallus gallus*) eggs. Furthermore, sperm nuclei were detected in the PVMs of 80 eggs from 39 exotic avian species, of which 73 eggs from 34 species were incubated for a minimum of 2–24 days, but had no embryonic development.

## Materials and methods

All experiments in this study were reviewed and approved by the San Diego Zoo Global (SDZG) IACUC (assurance# A3675-01) in compliance with the guidelines for the use of animals in research (OLAW/ARENA, 2002; National Research Council, 2011). The taxonomic classification and naming of birds is based on Sibley *et al.* (1988), Sibley and Monroe (1990) and Birdlife International (2014). Unincubated, fertilized chicken eggs were obtained from McIntyre Egg Ranch (Lakeside, CA, USA). Eggs of exotic species were received opportunistically from bird collections at SDZG breeding programmes, SeaWorld and the International Crane Foundation. Unless otherwise noted, all chemicals were purchased from Fisher Scientific (Pittsburg, PA, USA).

### Incubated, refrigerated and room-temperature chicken eggs

To determine the impact of temperature on PVM-bound sperm degradation and detection, eggs were incubated at 37.5°C and 60–70% humidity in a Roll-X forced air incubator (Lyon Electric Company, Chula Vista, CA, USA), stored at a room temperature of 20–22°C or refrigerated at 4°C. Incubated and room-temperature eggs were opened and examined for sperm in groups of five eggs at 5 day intervals over a period of 30 days. Five eggs were refrigerated and examined on day 30. Five control eggs were opened and examined immediately post-oviposition (fresh). Prior to incubation, the blastoderm was destroyed by cauterization through an eggshell window made with a belt sander, as described by Roe *et al*. (2013). This procedure localized damage without compromising the integrity of the PVM and prevented embryo development, which causes accelerated degradation of membrane-bound sperm due to changes in membrane structure during development (Jensen, 1969; Birkhead *et al.,* 2008). Eggshell windows were closed by melting Parafilm (Fisher Scientific) to the shell. Following treatment or upon receipt of an egg, the PVM was removed using fine scissors and forceps while floating the yolk in a bowl with deionized water, as previously described by Birkhead *et al.* (2008). In deteriorated eggs where the PVMs had lost integrity, as many pieces as were possible were collected by sifting with a pair of forceps. Excess yolk and albumen were removed from recovered membranes by rinsing with phosphate-buffered saline prior to staining. Although not tested during this project, it is not expected that membrane rinsing would influence the sperm counts. Membranes were stained with Hoechst 33342 for 10 min at room temperature in the dark and washed three times with phosphate-buffered saline. The PVMs were microscopically evaluated at ×200 magnification with a Zeiss AXIO Imager.A1 microscope with a UV florescence attachment (Zeiss, Thornwood, NY, USA). Eight to 10 photographs were taken at random areas of each recovered membrane. Blastoderm location was not taken into account, because this landmark was not always identifiable. Sperm counts from photogaphs at each time point and after each storage method were used to determine the average number of sperm per field of view (fov).

### Infected chicken eggs

To evaluate the detection of sperm in bacterially infected eggs, five chicken eggs were infected post-oviposition with 0.1 ml (1.5 × 10^8^ colony-forming units/ml) of cultured *Pseudomonas aeruginosa* ATCC 10145 (Microbiologics, St Cloud, MN, USA) through the previously described eggshell windows. *Pseudomonas aeruginosa* was selected as a model organism for this experiment because of its easily identifiable blue pigmented plaques and because it is commonly present in chicken egg infections (Lorenz *et al*., 1952; Leleu *et al*., 2011). Infected eggs were incubated until a bacterial plaque was visible by candling (2–7 days) at 37.5°C and 60–70% humidity in a forced air incubator. Infection was visually confirmed upon opening, and PVMs were recovered, stained and counted for sperm as described in the previous subsection. Eggs lacking signs of infection by candling after 7 days were excluded from the study to differentiate the effects of bacteria from those of incubation length.

### Exotic avian eggs

Avian eggs were stained to assess the feasibility of using PVM-bound sperm detection for breeding management of exotic species. Two chestnut-breasted malkoha (*Phaenicophaeus curvirostris*) eggs exhibiting very early signs of embryo development (stage 6; Hamburger and Hamilton, 1951) were included as a positive control for Hoechst staining. The PVMs of 72 exotic species were evaluated for the presence or absence of sperm. Perivitelline membrange-bound sperm nuclei were not detected in 33 of the 72 species and were excluded from Tables 1 and 2. Eggs from 19 species in the SDZG bird collection were analysed for management purposes (Table 3). These eggs from captive-breeding programmes either showed no sign of development following incubation or were removed prior to incubation. Confirmed sperm presence was defined as visual identification of two or more fluorescent, morphologically similar sperm heads on the PVM to prevent false positives from the counting of sperm-like debris. Sperm counts as described above for chicken eggs were not conducted for this portion of the study. Incubation data were provided by each breeding facility. The longest known incubation periods with confirmed sperm detection for each species are listed in Tables 1 and 2. Two eggs, one from from a mountain peacock pheasant and one from a great blue turaco, which were naturally infected with bacteria and/or fungus, were examined for PVM-bound sperm nuclei. Membrane pieces were recovered for Hoechst staining and DNA isolation.

**Table 1:** Detection of perivitelline membrane-bound sperm in various avian species

Order	Family	Genus, species, subspecies	Common name	Maximal no. of sperm/fov at longest incubation	Longest known incubation (days)	Conservation status
Struthioniformes	Apterygidae	*Apteryx mantelli*	Northern brown kiwi^a^	2	19	EN
Galliformes	Phasianidae	*Polyplectron inopinatum*	Mountain peacock-pheasant^a^	1	Unknown^b^	VU
		*Syrmaticus reevesii*	Reeves's pheasant^a^	9	18	VU
Anseriformes	Anatidae	*Cygnus melancoryphus*	Black-necked swan^a^	6	5	LC
		*Branta sandvicensis*	Hawaiian goose	1	<1	VU
		*Dendrocygna viduata*	White-faced whistling duck^a^	3	15	LC
		*Mergellus albellus*	Smew	1	6	LC
Piciformes	Ramphastidae	*Pteroglossus viridis*	Green aracari	3	2	LC
Coraciiformes	Alcedinidae	*Todiramphus chloris*	Collared kingfisher	1	5	NT
	Coraciidae	*Eurystomus orientalis*	Asian dollarbird	2	11	LC
	Phoeniculidae	*Phoeniculus purpureus*	Green wood hoopoe	7	7	LC
Cuculiformes	Cuculidae	*Phaenicophaeus curvirostris*	Chestnut-breasted malkoha	1	Unknown^c^	LC
		*Phaenicophaeus javanicus*	Red-billed malkoha	1	10	LC
Musophagiformes	Musophagidae	*Corythaeola cristata*	Great blue turaco^a^	4	38^b^	LC
Columbiformes	Columbidae	*Chalcophaps indica*	Emerald dove^a^	4	6	LC
		*Ducula rufigaster*	Purple-tailed imperial pigeon	12	10	LC
		*Geopelia cuneata*	Diamond dove	6	3	LC
		*Otidiphaps nobilis nobilis*	Green-naped pheasant pigeon	2	1	LC
		*Ptilinopus coronulatus*	Coroneted fruit dove	3	15	LC
		*Ptilinopus pulchellus*	Beautiful fruit dove	5	<1	LC

Abbreviations: EN, endangered; fov, field of view; LC, least concern; NT, near threatened; VU, vulnerable. ^a^DNA isolated and PCR amplified. ^b^Egg naturally infected. ^c^Stage 6 embryo.

**Table 2:** Detection of perivitelline membrane-bound sperm in various avian species

Order	Family	Genus species subspecies	Common name	Maximal no. of sperm/fov at longest incubation	Longest known incubation (days)	Conservation status
Gruiformes	Rallidae	*Rallus longirostris levipes*	Light-footed clapper rail^a^	2	22	EN
	Gruidae	*Anthropoides virgo*	Demoiselle crane	7	14	LC
		*Grus americana*	Whooping crane	1	11	EN
Ciconiiformes	Ciconiidae	*Ciconia stormi*	Storm's stork^a^	2	24	EN
		*Ephippiorhynchus senegalensis*	Saddle-billed stork	3	15	LC
		*Anastomus lamelligerus*	African openbill	2		LC
	Phoenicopteridae	*Phoenicopterus ruber*	American flamingo	1	13	LC
Falconiformes	Falconidae	*Polihierax semitorquatus*	Pygmy falcon	1	2	LC
Sphenisciformes	Spheniscidae	*Pygoscelis papua*	Gentoo penguin	5	18	NT
Passeriformes	Corvidae	*Corvus hawaiiensis*	Hawaiian crow^a^	1	10	EW
	Estrildidae	*Poephila acuticauda*	Long-tailed finch	1	1	LC
	Laniidae	*Lanius ludovicianus mearnsi*	San Clemente loggerhead shrike^a^	1	24	CR
	Paradisaeidae	*Lophorina superba*	Superb bird-of-paradise	1	12	LC
		*Paradisaea raggiana*	Raggiana bird-of-paradise	1	12	LC
	Pycnonotidae	*Hypsipetes leucocephalus*	Asian black bulbul	3	8	LC
	Sturnidae	*Coccycolius iris*	Emerald starling	1	4	DD
		*Scissirostrum dubium*	Finch-billed myna	1	8	LC
	Timaliidae	*Garrulax courtoisi*	Blue-crowned laughingthrush	2	<1	CR
	Fringillidae	*Loxioides bailleui*	Palila	2	11	CR

Abbreviations: CR, critcally endangered; DD, data deficient; EN, endangered; EW, extinct in the wild; fov, field of view; LC, least concern; NT, near threatened; VU, vulnerable. ^a^DNA isolated and PCR amplified.

**Table 3:** Management-requested perivitelline membrane-bound detection in San Diego Zoo Global avian collection

Family	Species	Common name	No. of pairs	No. of eggs examined	No. of positive eggs (%)	Reason for testing
Apterygidae	*Apteryx mantelli*	Northern brown kiwi	2	2	1 (50)	Male fertility test, repeated non-developing eggs
Anatidae	*Branta sandvicensis*	Hawaiian goose	1	1^a^	1 (100)	Check for pair fertility, thin eggshell, female nutrition issue
	*Anser indicus*	Bar-headed goose	1	2^a^	0 (0)	Check male fertility
Alcedinidae	*Todiramphus cinnamominus*	Guam kingfisher	1	1	0 (0)	Check pair fertility
Rallidae	*Rallus longirostris levipes*	Light-footed clapper rail	1	3	3 (100)	Suspected parent incubation failure, test fertility
Gruidae	*Anthropoides paradiseus*	Blue crane	1	2	0 (0)	Check pair fertility
	*Anthropoides virgo*	Demoiselle crane	2	2	1 (50)	Check pair fertility pre-AI. check success of AI
	*Rhynchopsitta pachyrhyncha*	Wattled crane	1	1	0 (0)	Check pair fertility
	*Grus japonensis*	Red-crowned crane	1	1	0 (0)	Check pair fertility pre-AI
Ciconiidae	*Anastomus lamelligerus*	African openbill	2	13	2 (15.4)	Check pair fertility
Falconidae	*Polihierax semitorquatus*	African pygmy falcon	1	8	0 (0)	Check pair fertility
Spheniscidae	*Pygoscelis adeliae*	Adelei penguin	3	3	0 (0)	Check pair fertility
	*Pygoscelis papua*	Gentoo penguin	10	10	1 (10)	Check pair fertility
Psittacidae	*Anodorhynchus hyacinthinus*	Hyacinth macaw	1	4	0 (0)	Check pair fertility
	*Rhynchopsitta pachyrhyncha*	Thick-billled parrot	1	1^b^	0 (0)	Check pair fertility
Corvidae	*Corvus hawaiiensis*	Hawaiian crow	18	53	11 (20.8)	Results are incorporated into a large species-wide evaluation programme to maximize reproductive output and genetic diversity, while minimizing inbreeding
Laniidae	*Lanius ludovicianus mearnsi*	San Clemente loggerhead shrike	15	34	5 (14.7)	Results are used in the management of captive breeding pairs to maximize reproductive output and genetic diversity, while minimizing inbreeding
Timaliidae	*Garrulax courtoisi*	Blue-crowned laughingthrush	1	4	0 (0)	Check pair fertility
Oriolidae	*Oriolus auratus*	Golden oriole	1	2	0 (0)	Check pair fertility

Abbreviation: AI, artificial insemination. ^a^Unincubated eggs. ^b^Not including egg containing early embryonic death.

### DNA isolation and PCR

DNA was isolated from sperm embedded in the PVM, as previously described by Carter *et al.* (2000), with modifications. Briefly, the PVMs were incubated in extraction buffer (0.2 m Tris, 0.1 m EDTA, 1% sodium dodecyl sulfate and 0.1% proteinase K) at 37°C overnight. Samples were diluted 1:1 with phenol:chloroform:isoamyl alcohol (IAA) (25:24:1), vortexed and centrifuged at 6000***g*** for 5 min. The aqueous phase was diluted 1:1 with chloroform:IAA (24:1) centrifuged at 6000***g*** for 5 min, followed by precipitation of the aqueous phase by adding 0.1 m NaCl and two volumes of 100% ethanol before storing at −80°C overnight. The samples were centrifuged at 21 000***g*** for 30 min at 4°C, followed by 100 and 75% ethanol washes and resuspension in 20 μl Tris-EDTA buffer.

Polymerase chain reactions were performed only on DNA isolated from membranes verified as sperm positive by Hoechst staining, as well as on known negative controls. Primers used were P2 (5′-TCTGCATCGCTAAATCCTTT-3′) and P8 (5′-CTCCCAAGGATGAGRAAYTG-3′) as described by Griffiths *et al.* (1998), which produce a single Z band in males and separate Z and W bands in females. These primers are capable of amplifying DNA in all species tested, although they may not be able to discriminate between Z and W alleles in some species *(*Griffiths *et al.*, 1998; Huynen *et al*., 2002; Jensen *et al*., 2003).

The PCR amplifications were carried out in 15 μl reactions containing 13.5 μl Platinum Blue PCR SuperMix (22 U/ml *Taq*, 22 mm Tris–HCl, 55 mm KCl, 1.65 mm MgCl_2_ and 220 µm of each dNTP; Invitrogen, Carlsbad, CA, USA), 200 nm of each primer and 1 μl gDNA. Polymerase chain reactions were performed on a GeneAmp 9700 thermo-cycler (Life Technologies, Grand Island, NY, USA) in the following three steps: denaturation at 95°C for 2 min, followed by amplification for 30 cycles at 95°C for 30 s, 48°C for 30 s and 72°C for 30 s, followed by a final extension at 72°C for 5 min. The PCR products were separated on 1.5% agarose gels and visualized on a Kodak Electrophoresis Documentation and Analysis System 120 (Kodak, Rochester, NY, USA). Polymerase chain reactions that did not exhibit detectable products were re-amplified (double PCR) using the products of the first PCR as template DNA.

## Results

### Hoechst staining for morphology

**Figure 1:**
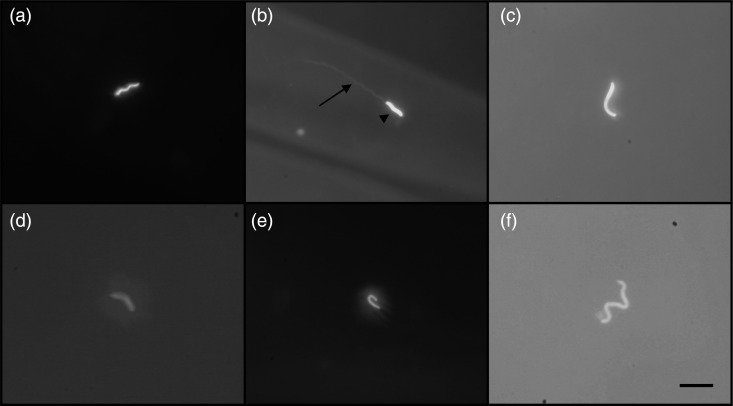
Sperm bound on perivitelline membranes stained with Hoechst 33342. (a) Hawaiian crow (*Corvus hawaiiensis*). (b) Finch-billed myna (*Scissirostrum dubium*). (c) Northern brown kiwi (*Apteryx mantelli*). (d) Storm's stork (*Ciconia stormi*). (e) Collared kingfisher (*Todiramphus chloris*). (f) Green-naped pheasant pigeon (*Otidiphaps nobilis nobilis*). Arrowhead indicates sperm head; arrow, sperm tail. Bar represents 10 μm.

Sperm morphology varied between species, but all possessed readily identifiable sperm heads (Fig. 1). Sperm head morphology did not appear to vary greatly between individuals of a species (data not shown).

### Chicken eggs

**Figure 2:**
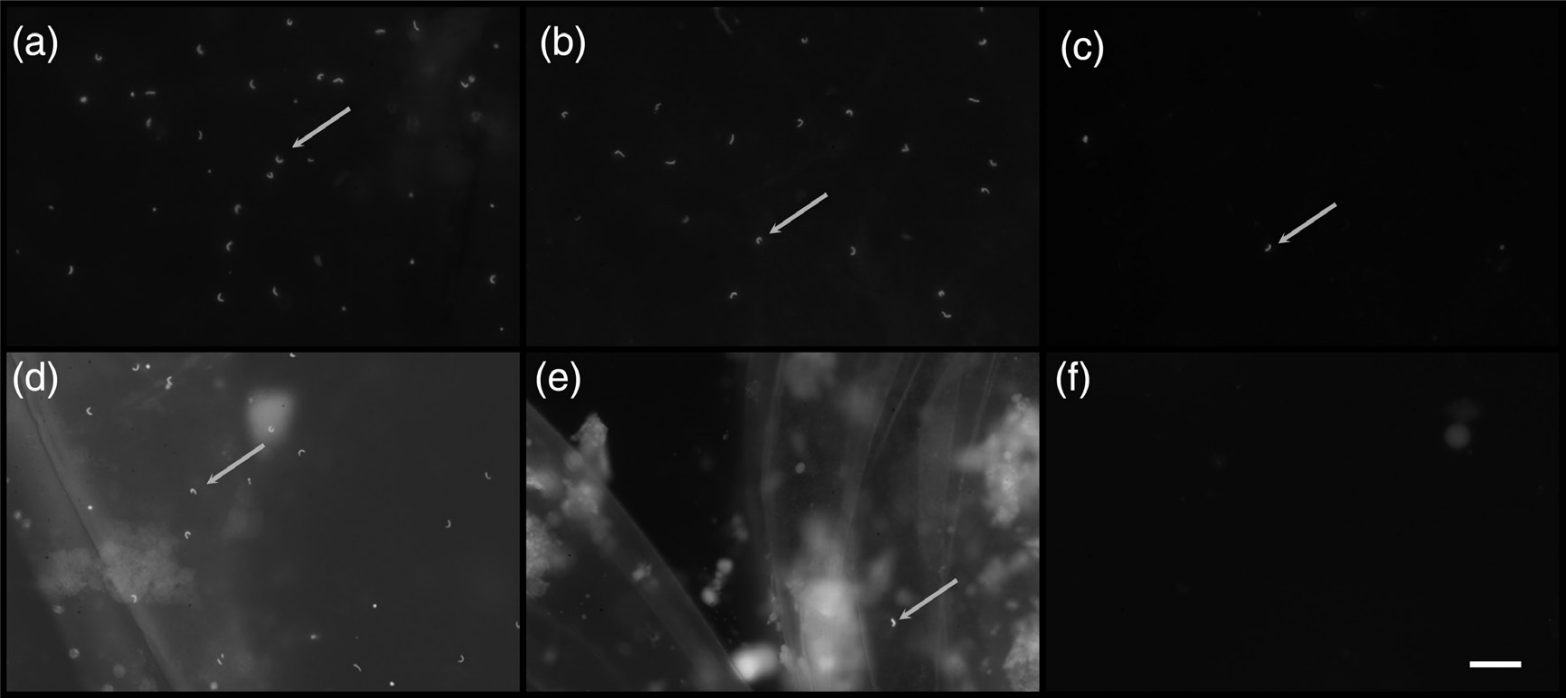
Detection of sperm bound on perivitelline membranes by Hoechst 33342 staining in fresh chicken egg (a), chicken eggs stored at room temperature (20–22°C) for 15 (b) and 30 days (c), chicken eggs stored at refrigeration temperature (4°C) for 30 days (d) and chicken eggs stored at incubation temperature (37.5°C) for 15 (e) and 30 days (f). Arrows indicated sperm heads. Bar represents 50 μm in all panels.

**Figure 3:**
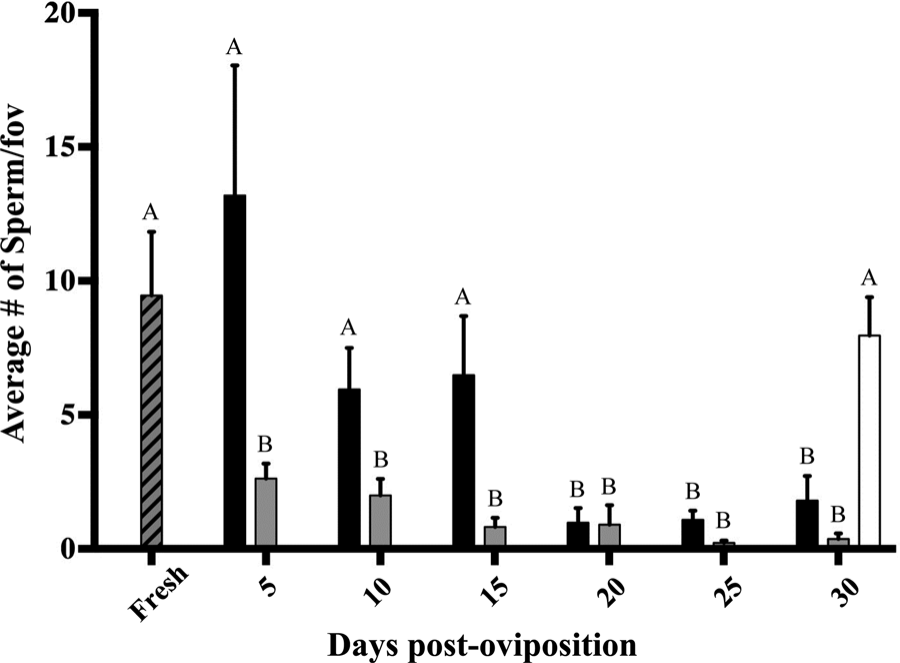
Detection of perivitelline membrane-bound sperm by Hoechst 33342 staining in fresh eggs (grey hatched bar), room-temperature-stored eggs (black bars), incubated (37°C) eggs (grey bars) and refrigerated eggs (white bar). Letters denote significant difference (*P* < 0.05) in number of sperm between fresh and all other treatments.

Sperm counts in room-temperature and incubated chicken eggs decreased significantly over time. Fresh PVMs had 9.45 ± 2.37 (mean ± SEM) sperm per fov, while room-temperature eggs had an average of 13.2 ± 4.85 sperm per fov on day 5, 5.95 ± 1.55 on day 10, 6.49 ± 2.20 on day 15, 0.98 ± 0.54 on day 20, 1.08 ± 0.34 on day 25 and 1.80 ± 0.92 sperm per fov on day 30 (Figs 2 and 3).

Incubated eggs had an average of 2.62 ± 0.57 sperm per fov on day 5, 2.00 ± 0.61 sperm per fov on day 10, 0.82 ± 0.34 on day 15, 0.90 ± 0.73 on day 20, 0.22 ± 0.09 on day 25 and 0.37 ± 0.21 sperm per fov on day 30 (Figs 2 and 3). Two-way ANOVA showed a significant decrease in sperm counts per fov for eggs stored at both room temperature and incubation temperature [F(1, 55) = 11.1, *P* = 0.0016] and for number of days [*F*(6, 55) = 7.58, *P* < 0.0001] compared with fresh eggs (Fig. 3).

Refrigerated egg sperm counts were not statistically different from those of fresh eggs, with an average of 7.96 ± 1.43 sperm per fov on day 30 (*P* = 0.60, *t* = 0.54, d.f. = 8; Figs 2 and 3).

### Infected eggs

**Figure 4:**
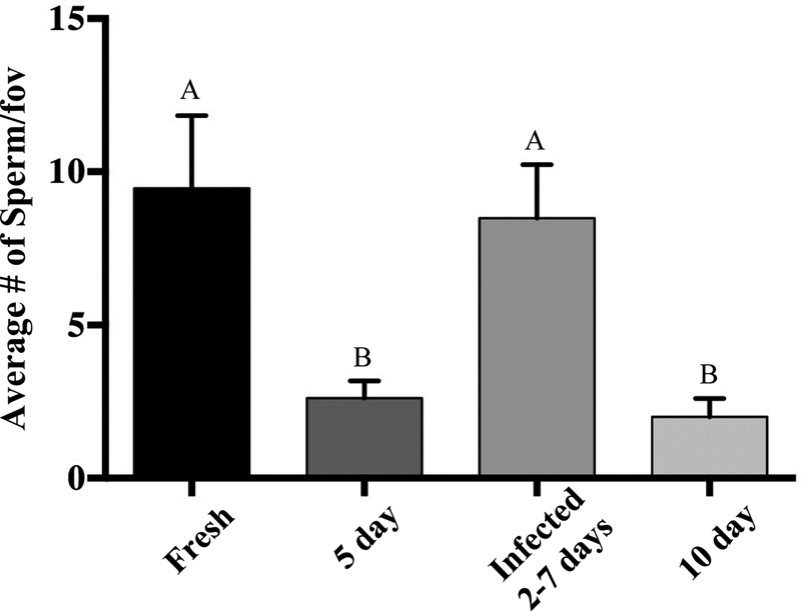
Detection of perivitelline membrane-bound sperm by Hoechst 33342 staining in artificially infected chicken eggs incubated until large microbial plaque formation (2–7 days). Letters denote significant difference (*P* < 0.05) in number of sperm between infected and fresh, 5 and 10 day incubated eggs.

**Figure 5:**
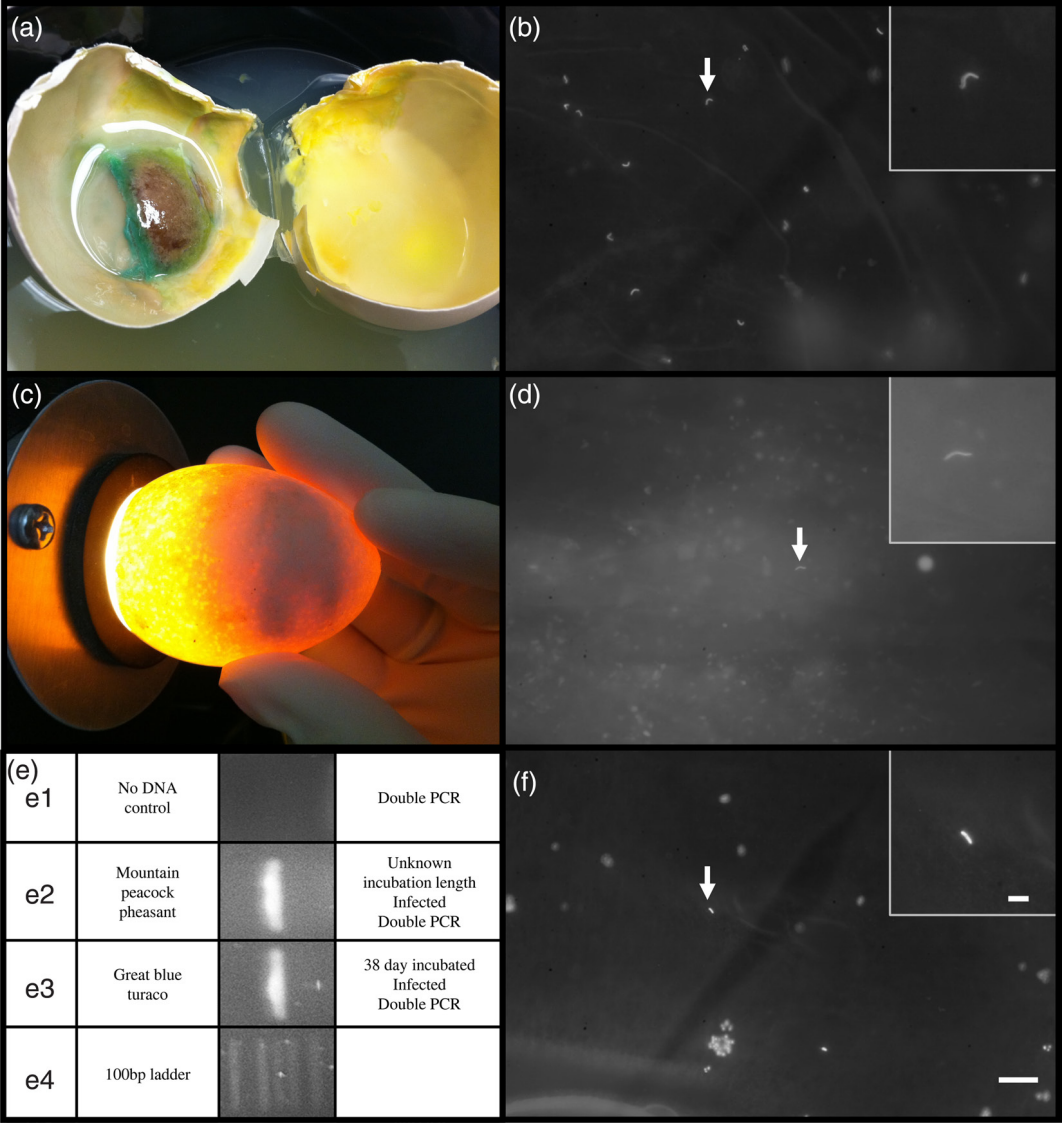
Detection of perivitelline membrane-bound sperm by Hoechst 33342 staining in artificially infected chicken egg (a and b) and naturally infected mountain peacock-pheasant egg (c, d and e2) and great blue turaco egg (e3 and f). Insets represents increased magnification. (e) Polymerase chain reaction amplification of presumed sperm DNA from microbially infected vitelline membranes. Bar represents 50 or 10 μm (insets). Great blue turaco egg, incubation data unknown, ambient temperature for up to 38 days.

Fresh chicken eggs infected with *P. aeruginosa* had an average of 8.48 ± 1.76 sperm per fov following 2–7 days of incubation, which was not statistically different from fresh eggs (*P* = 0.75 *t* = 0.33 d.f. = 8). Although infected eggs were incubated at 37.5°C for 2–7 days, there was a statistical difference between the number of sperm per fov in infected and day 5 (*P* = 0.0131, *t* = 3.2, d.f. = 8) and day 10 incubated eggs (*P* = 0.0083, *t* = 3.48, d.f. = 8; Fig. 4). Sperm nuclei were detected by Hoechst staining and by PCR in infected great blue turaco and mountain peacock pheasant eggs (Fig. 5). Visual detection is greatly affected by the amount of bacteria or fungal hyphae present, because the bacterial and fungal DNA also fluoresces, obscuring the sperm fluorescence.

### Exotic avian eggs

During this study, 78 eggs were determined to have two or more sperm per PVM; of these, 71 were incubated for >48 h with no visible embryonic development. The 78 eggs with two or more detectable and identifiable PVM-bound sperm represent 38 species in 13 orders (Tables 1 and 2). Eggs that had fewer than two sperm per PVM were disregarded in this study to minimize the potential for false positives. Of the 19 species analysed for SDZG management, eight species were positive for PVM-bound sperm (Table 3). Table 4 shows management decisions and re-pairings of specific San Clemente loggerhead shrikes based, in part, on the results of PVM staining information.

**Table 4:** Perivitelline membrane-bound sperm detection in San Clemente loggerhead shrike (*Lanius ludovicianus mearnsi*) for management of specific pairs

Pairing sire/dam	Number of eggs examined	Number of sperm-positive PVMs (%)	Management comments	Conclusions
2635/1510	1	0 (0)	Other eggs in clutch developed	Pair fertility confirmed
355/2046	4	0 (0)	Male (355) suspected of being too old for mating, female (2046) re-paired with male (2564) following PVM analysis. Other eggs in 2564/2046 pair clutch developed	Male retired from breeding programme, female re-paired according to genetic schedule
2564/2046	1	0 (0)		
1118/2046	2	0 (0)	Female re-pair, no development in clutch, both proven breeders	Pair behavioural incompatibility, female re-paired according to genetic schedule
407/2046	5	0 (0)	Female re-pair, no development in clutch, both proven breeders	Pair behavioural incompatibility, female re-paired according to genetic schedule
2555/987	2	0 (0)	Other eggs in clutch developed, 2012	Pair fertility confirmed
	3	1 (33)	Other eggs in clutch developed, 2013	Pair fertility confirmed
2387/1856	1	1 (100)	Single-egg clutch, second clutch, no development	Pair fertility good, possible inappropriate incubation behaviour
2341/698	1	0 (0)	Other eggs in clutch developed	Pair fertility confirmed
632/1255	5	0 (0)	No development in clutch, both proven breeders	Pair behavioural incompatibility
1502/1296	1	1 (100)	Other eggs in clutch developed	Pair fertility confirmed
	1	0 (0)	Male removed 8 days before second clutch, no development	Female sperm storage likely not to extend to 8 days
792/1074	1	1 (100)	Other eggs in clutch developed	Pair fertility confirmed
407/2046	5	0 (0)	No development in clutch, both proven breeders	Pair behavioural incompatibility
1522/2526	1	1 (100)	One-egg clutch, female abandoned nest, second clutch developed	Pair fertility good, possible inappropriate incubation behaviour
471/1700	5	0 (0)	No development in clutch, both proven breeders	Pair behavioural incompatibility

Abbreviation: PVM, perivitelline membrane.

### Polymerase chain reaction amplification

**Figure 6:**
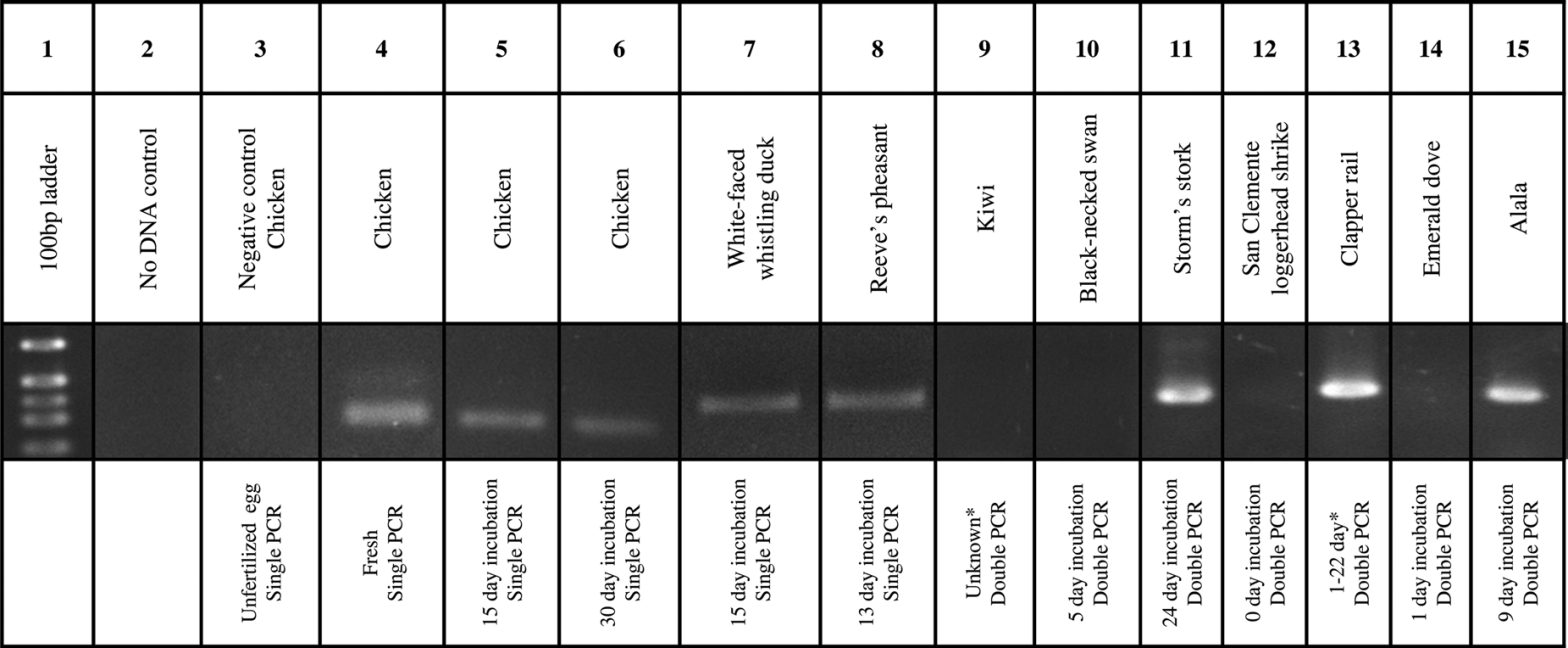
Detection of perivitelline membrane-bound sperm by single or double PCR in incubated eggs from multiple species. Lanes 1–8 represent single PCRs, while lanes 9–15 represent double PCRs. *Eggs that were parent incubated for an unknown length of time. All eggs used in the PCRs were verified positive by visual sperm detection.

Polymerase chain reaction amplicons were detected in fresh, 15 day and 30 day incubated chicken eggs, with an apparent decrease in band intensity from fresh to 30 day incubated (Fig. 6). Perivitelline membranes from unfertilized chicken eggs did not produce any amplicons.

The PCR detection of sperm was successful in the white-faced whistling duck (*Dendrocygna viduata*), Reeve's pheasant (*Symaticus reevesii*), Storm's stork (*Ciconia stormi*), light-footed clapper rail (*Rallus longirostris levipes*) and Hawaiian crow (*Corvus hawaiiensis*; Fig. 6) eggs. The PCR detection of DNA was not successful in the Northern brown kiwi (*Apteryx mantelli*), black-necked swan (*Cygnus melacoryphus*), San Clemente loggerhead shrike (*Lanius ludovicianus mearnsi*) and emerald dove (*Chalcophas indica*; Fig. 6), although at least two sperm per PVM were detected by Hoechst staining. In addition, DNA was amplified from naturally infected mountain peacock-pheasant (*Polyplectron inopinatum*) and great blue turaco (*Corythaeola cristata*) eggs (Fig. 5).

## Discussion

### Degradation during incubation

Exotic eggs are often incubated for 10–20 days or longer before the decision is made to remove them from incubation in the absence of development. It was therefore important to demonstrate the ability of this PVM-bound sperm detection technique to identify sperm consistently within this time frame. This study demonstrated that at least two sperm per fov could be detected on the PVM for up to 24 (exotic) and 30 days (chicken). The significant decrease in sperm nuclei detected over time is the result of DNase I and II activity in the cytoplasmic layer adhering to the PVM (Stepińska and Olszańska, 2003) as a defense against polyspermy. However, there may be species-specific differences in the rate of PVM-bound sperm degradation; a study by Wishart (1997) demonstrated that turkey sperm nuclei decondense faster than those of chickens. Therefore, failure of detection of sperm nuclei after initiation of incubation, and especially after prolonged incubation, may not indicate an absence of sperm in the infundibulum at the time of ovulation, resulting in a false-negative result.

### Expected egg fertility

When assessing the presence of PVM-bound sperm for management, this technique should be viewed as qualitative and not quantitative unless DNase activity and minimal sperm fertilization concentration have been determined. Therefore, the density or absolute number of sperm are not useful quantities unless the minimal number of PVM-bound sperm necessary for 0 and 100% fertilization rates are known. This is obviously not feasible for routine management of exotic species collections, because it involves artificial insemination with known concentrations of sperm, and correlation of these doses with the percentage of fertile eggs and PVM-bound sperm counts. The DNase-caused sperm degradation is rapid during incubation, such that by 10–20 days of incubation, when non-developing eggs are routinely removed from incubation, the sperm count would be difficult to correlate with the number of sperm present in the infundibulum.

It is possible that the sperm concentration could be low enough to result in failure of fertilization, but still be detectable by staining. However, in the zebra finch (*Taeniopygia guttata*), approximately 30 total PVM-bound sperm resulted in 100% fertility (Birkhead and Fletcher, 1998). Consequently, it is unlikely that two sperm (our positive search criteria) would be detected in incubated eggs with a low enough sperm count to be unfertilized, considering the decrease of detectable sperm per ×200 fov from 9.5 ± 2.4 in fresh to 2.0 ± 0.6 by 10 days and 0.9 ± 0.7 by 20 days of incubation, in chickens.

A false-negative PVM evaluation could occur following incubation if sperm counts were low enough to degrade completely by 10–20 days of incubation. Such a result may or may not be different functionally from a ‘no sperm’ designation, i.e. subfertile sperm production. Thus, negative PVM results from incubated eggs should be confirmed by staining PVMs from freshly laid eggs to preclude false negatives caused by the effect of incubation on sperm degradation/detection.

For management purposes, we assume the detection of two sperm on the PVM of incubated eggs to indicate that sufficient sperm were present at the time of ovulation to fertilize 100% of eggs. The presence of PVM-bound sperm, but without embryo development, could be the result of a non-functional oocyte (i.e. abnormal meiosis), a fertilization problem (lack of penetration or pro-nuclear formation) or pre-/post-oviposition blastoderm death (lack of incubation, genetic or developmental problem), rather than a male sperm production or quality issue. If the reproductive history of either or both the sire and dam are known, most of these causes of infertility could be eliminated, resulting in better pair management.

### Polymerase chain reaction

The PCR detection of sperm was not possible in very large or very small eggs with low sperm counts, probably due to the low concentration of DNA following phenol–chloroform isolation and precipitation, making visual verification more reliable. The PCR could potentially be contaminated with blastoderm embryo DNA, which would not be distinguishable from sperm DNA if the embryo was male. This would not interfere with fertility assessment, but could influence sperm genotyping results or blastoderm sexing.

### Bacterial degradation

Bacterial (*P. aeruginosa)* infection following 2–7 days of incubation did not decrease sperm counts significantly compared with fresh eggs. However, sperm counts on incubated non-infected eggs were significantly less than on fresh eggs by 5 days of incubation. A highly speculative explanation could be a microbial block of endogenous DNases, perhaps as a microbial defense mechanism during early infection.

Even though the yolk membrane often disintegrates during infection, a degraded egg does not necessarily preclude the detection of sperm nuclei. In fact, PVM-bound sperm were detected in mountain peacock-pheasant and great blue turaco eggs, despite recovery of only small fragments of membrane covered in fungal hyphae. However, the possibility of a false-negative result increases greatly with incubation and might warrant the sacrifice of a fresh egg for a more accurate assessment. In summary, PVM-bound sperm detection in highly infected or degraded eggs is useful only if the result is positive or can be verified using fresh eggs from the pair.

### Artificial insemination and exotic pair management

Information derived from PVM-bound sperm detection is currently used in the management of pairs from 19 species in the SDZG collection. Perivitelline membrane staining is a useful tool, which, in combination with behavioural observations and past breeding history, can be used to evaluate potential causes of reproductive failures.

One example of the successful use of this technique is for pairs that do not successfully breed naturally and are managed by artificial insemination. Following artificial insemination, a lack of visibly fertile eggs (i.e. initiation of embryonic development) could be due to several factors, such as poor semen quality or handling, poor insemination technique or timing, or female reproductive problems. Perivitelline membrane-bound sperm detection can, in addition to the sire and dam history, establish the cause of developmental failure. The bird department of SDZG is currently using this technique to verify infertility of eggs from candidates for artificial insemination and to verify egg fertilization (of non-developing eggs) following artificial insemination of red-crowned cranes (*Grus japonensis*) and Demoiselle cranes (*Anthropoides virgo*; Table 3). Pairs of the San Clemente loggerhead shrike, a species under management by SDZG (and partners), with eggs that do not exhibit any signs of embryological development are stained for PVM-bound sperm. Decisions for re-pairings or removal of eggs for artificial incubation are made based on the PVM-bound sperm data in combination with behavioural observations, assuming that a lack of sperm in unincubated eggs indicates male infertility or pair incompatibility, while sperm presence may indicate inappropriate incubation behaviour.

### Summary

This study demonstrated the practicality of using the detection of PVM-bound sperm nuclei for the management of a broad range of exotic species. Presumed fertility and sperm function can be determined by detection of PVM-bound sperm nuclei visually or by PCR, in fresh eggs, in eggs incubated for up to 24 days and in infected eggs. However, as PVM-bound sperm appear to degrade at temperature- and species-specific rates, eggs should be removed from incubation and tested as soon as a lack of embryological development is confirmed by candling.

At SDZG, staining of PVM-bound sperm is currently used as a management tool to evaluate the fertility of pairs representing 19 species in our breeding programme, including verification of functional sperm post-artificial insemination of two crane species. While eggs are often analysed shortly after being removed from incubation, there are circumstances where analysis is not possible immediately. As the PVM becomes increasingly fragile during incubation, especially when infected, the difficulty of recovery of the PVM increases, and it is important that eggs are stored at 4°C to prevent further PVM degradation, sperm loss or excessive microbial growth.

## Funding

This work was in part supported by a grant from the NOJ-Foundation [to T.J.].
